# Prognostic value of serum NLR, PLR, P53, K67 level in lymph node metastasis of early gastric cancer

**DOI:** 10.5937/jomb0-55634

**Published:** 2025-07-04

**Authors:** Xin Long, Yingxi Shi, Feifei Li, Zhaojun Wang, Ying Wang

**Affiliations:** 1 Sichuan Integrative Medicine Hospital, Department of Medical Oncology, Chengdu, China; 2 Chengdu University of Traditional Chinese Medicine, Clinical School of Medicine, Chengdu, China; 3 Sichuan Academy of Medical Sciences & Sichuan Provincial People's Hospital, School of Medicine UESTC, Oncology Center, Chengdu, China; 4 Dazhou Integrated TCM & Western Medicine Hospital, Department of Medical Oncology, Chengdu, China

**Keywords:** serum NLR, PLR, P53, K67, early gastric cancer, pathological features, lymph node metastasis, risk factors, NLR u serumu, PLR, P53, K67, rani karcinom 'eluca, patološke karakteristike, metastaze limfnih čvorova, faktori rizika

## Abstract

**Background:**

To explore the predictive value of relevant detection indexes and pathological serum NLR, PLR, P53, and K67 levels in lymph node metastasis (LNM) in patients with early gastric cancer (EGC) after radical surgery.

**Methods:**

Clinical data of EGC patients (297 cases, all of whom underwent radical gastrectomy for gastric cancer) admitted to Sichuan Integrative Medicine Hospital from March 2019 to March 2024 were retrospectively included. The clinical data and pathological results were recorded and compared, and the related predictive factors were analysed.

**Results:**

There were 43 cases (14.48%) of postoperative LNM among the 297 EGC patients. The average number of lymph nodes detected in the LNM (-) group was 28.35 ± 8.23, which was lower than in the LNM (+) group (33 ± 15), *P* < 0.01. Binary multivariate logistic regression analysis identified the following as significant predictors of postoperative LNM in EGC patients: tumour size (OR: 2.582, 95% CI: 1.205-5.534), depth of invasion (OR: 2.953, 95% CI: 1.327-6.573), vascular invasion (OR: 2.724, 95% CI: 1.241-5.976), neuroaggression (OR: 2.681, 95% CI: 1.139-6.311), differentiation type (OR: 2.426, 95% CI: 1.140-5.119), and P53 (OR: 3.133, 95% CI: 1.183-8.301), P<0.05. The area under the ROC curve (AUC) for the model based on these indexes was 0.801. Compared with the LNM (-) group, the LNM (+) group had a lower overall survival rate at 1 and 2 years (P<0.05).

**Conclusions:**

Clinically relevant detection indexes and pathological P53 levels in patients after EGC radical surgery have a good predictive effect on the occurrence of LNM, which can assist in formulating scientific and reasonable clinical treatment plans.

## Abstract (sr)

**Uvod:**

Cilj je bio da se ispita prognostička vrednost relevantnih indeksa detekcije i patolo{kih nivoa NLR, PLR, P53 i K67 u serumu kod metastaza limfnih čvorova (LNM) kod pacijenata sa ranim karcinomom želuca (EGC) nakon radikalne operacije.

**Metode:**

Ura|ena je retrospektivna analiza kliničkih podataka pacijenata sa EGC-om (297 slučajeva, svi su podvrgnuti radikalnoj gastrektomiji zbog karcinoma želuca) primljenih u Bolnicu za integrativnu medicinu u Sičuanu od marta 2019. do marta 2024. Klinički podaci i patološki rezultati su zabeleženi i upoređeni, a analizirani su i povezani prognostički faktori.

**Rezultati:**

Od ukupno 297 pacijenata sa EGC-om, 43 slučaja (14,48%) su imala postoperativne LNM. Prosečan broj detektovanih limfnih čvorova u grupi LNM (-) bio je 28,35 ± 8,23, što je manje u odnosu na grupu LNM (+) (33±15), P<0,01. Binarnom multivarijantnom logisti~kom regresionom analizom identifikovani su sledeći značajni prediktori postoperativnih LNM kod pacijenata sa EGC-om: veličina tumora (OR: 2,582, 95% CI: 1,205-5,534), dubina invazije (OR: 2,953, 95% CI: 1,327-6,573), vaskularna invazija (OR: 2,724, 95% CI: 1,241-5,976), neuroadresija (OR: 2,681, 95% CI: 1,139-6,311), tip diferencijacije (OR: 2,426, 95% CI: 1,140-5,119) i P53 (OR: 3,133, 95% CI: 1,183-8,301), P<0,05. Površina ispod ROC krive (AUC) za model zasnovan na ovim parametrima iznosila je 0,801. U pore|enju sa grupom LNM (-), grupa LNM (+) imala je nižu stopu preživljavanja na 1 i 2 godine (P<0,05).

**Zaključak:**

Klinički relevantni indeksi detekcije i patološki nivoi P53 kod pacijenata nakon radikalne operacije EGC-a imaju dobru prognostičku vrednost za pojavu LNM, što može pomoći u formiranju naučno utemeljenih i racionalnih kliničkih planova lečenja.

## Introduction

Gastric cancer is a common malignant tumour that poses a significant threat to human survival [1]. Due to the atypical early symptoms of the disease, most patients with early gastric cancer (EGC) can achieve a cure through radical surgery [2]. EGC is characterised by tumour invasion confined to the mucosa and submucosa, with or without regional lymph node metastasis (LNM), enabling a high likelihood of recovery [3].

Although surgery for EGC is generally effective, some patients experience relapse and die within ashort period post-surgery, with LNM being a critical factor influencing and predicting the prognosis of EGC [4]. Studies have shown that patients with EGC and LNM have a significantly lower 5-year survival rate [5]. Thus, accurate preoperative assessment of LNM is essential for optimising clinical decision-making.

Many studies have identified risk factors associated with LNM in EGC, including tumour location, size, depth of invasion, gross classification, ulceration, differentiation type, vascular invasion, and nerve invasion [6] [7]. Numerous molecular markers are also considered effective predictors of LNM and gastric cancer prognosis. The P53 and Ki67 proteins, commonly studied in clinical oncology, have been shown to correlate with the degree of LNM and the depth of tumour invasion [8] [9].

Clinical guidelines for managing EGC after radical resection in China are based on international recommendations. This reliance may be attributed to the limited number of studies examining predictive factors for LNM following radical resection of EGC in China. This paper investigates the predictive value of relevant detection indices and pathological P53 levels for LNM after EGC radical surgery, aiming to provide insights into improving prognosis and developing targeted intervention strategies.

## Materials and methods

### Basic information

Clinical data of EGC patients (297 cases, all of whom received radical gastrectomy for gastric cancer) admitted to Sichuan Integrative Medicine Hospital from March 2019 to March 2024 were retrospectively included.

Inclusion criteria:: ① EGC was confirmed and rechecked according to WHO criteria [10]; ②Radical gastrectomy and lymph node dissection (all patients), and no antitumor therapy was performed before surgery; ③ All patients underwent gastroscopy in our hospital before an operation and had clear and complete imaging data; ④ Clinical data and follow-up data are complete. Excluded labels:① the presence of primary cancer in other locations;② In the past, there is no resection operation; ③ Advanced gastric cancer; ④ Complicated with other systemic diseases; ⑤ The evaluation of lesion morphology was affected by endoscopic biopsy performed in another hospital.

### Methods

Clinical and pathological data were collected for all patients through the pathology electronic record system. These data included sex, age, tumour size, general classification, depth of infiltration, nerve invasion, tumour location, vascular invasion, signet-ring cells, differentiation types, ulcers, and other relevant factors. Laboratory indicators included the neutrophilto- lymphocyte ratio (NLR) and platelet-to-lymphocyte ratio (PLR). The molecular markers analysed were P53 and K67.

– Tumour Site: The greater and lesser curvatures of the stomach were divided into three equal parts: lower (angle, antrum, and pylorus), middle (body of the stomach), and upper (fundus and cardia).
– Tumour Size: The maximum tumour diameter was calculated.
– General Classification of Tumour: Based on the Paris classification, tumours were categorised as elevated (0-I), flat (0-II), or depressed (0-III).
– Differentiation Type: Tumours were classified as differentiated or undifferentiated according to the 2014 guidelines of the Japanese Gastric Cancer Society [11].
– Ulcer Criteria: Lesions or scars were considered ulcers if they reached or exceeded the depth of the muscularis mucosa.
– Depth of Tumour Invasion: Tumour invasioninvolves the mucosal layer or the submucosa. The submucosa was further divided into the superficial and deep submucosal layers, depending on whether the maximum vertical distance from the deep muscularis mucosa layer to the lesion exceeded 500 μm.
– Vascular Metastasis: This included both lymphatic and microvascular metastases.
– Lymph Node Status: Patients were classified into the LNM-positive (LNM [+]) group and the non-LNM (LNM [-]) group based on postoperative pathology results.

### Follow-up

All patients were followed up for two years. For those with a follow-up period of less than two years, the survival outcomes were analysed and predicted based on their most recent examination results. The 1-year and 2-year overall survival (OS) rates of the Non-LNM (-) group and the LNM (-) group were compared.

### Statistical methods

All data were recorded using Excel 2007 and analysed with SPSS 25.0 software. Categorical variables were expressed as [n (%)], while continuous variables were presented as (mean ± SD). Statistical analyses included the x^2^ test for categorical variables and the T-test for continuous variables. Univariate and multivariate logistic regression analyses were conducted to identify predictive factors for postoperative LNM in EGC patients, and their predictive value was evaluated. The significance level for all statistical tests was set at α=0.05.

## Results

### Lymph node metastasis

**Figure 1:**
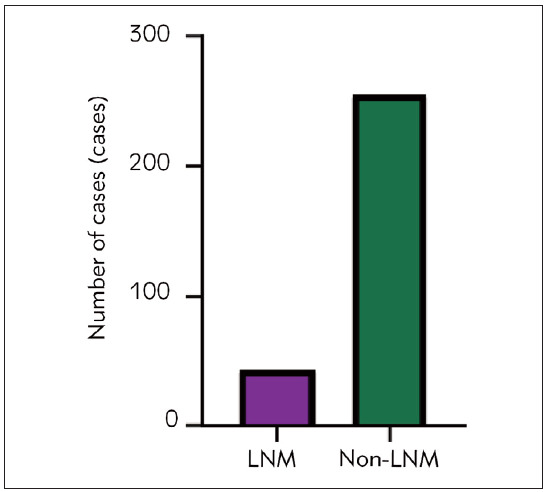
Prediction of intraoperative risk column chart.

Among 297 EGC patients, 43 (14.48%) developed LNM after radical resection, Figure 1.

### Number of LNM

**Figure 2:**
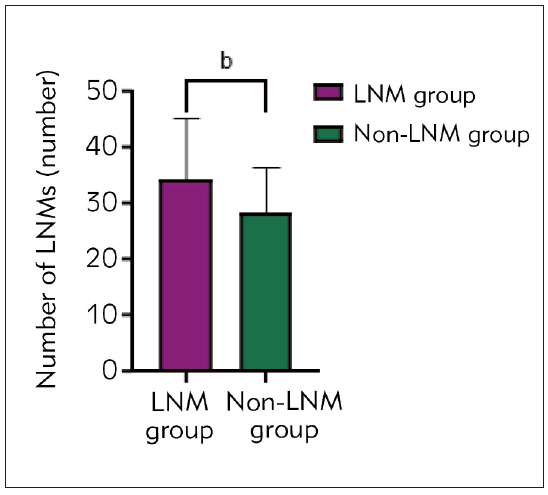
Comparison of LNM number between Non-LNM (-) group and LNM (+) group.

The LNM (-) group’s average number of lymph nodes detected was (28.35±8.23), which is lower than the non-LNM (+) group (33±15), P<0.01, Figure 2.

### Univariate analysis

Invasion depth, tumour size, vasculature and nerve invasion, differentiation type, P53, and Non-LNM (-) group were compared (all P<0.05). Other factors were compared between the two groups (P>0.05); see Table 1.

**Table 1:** Univariate analysis.

Clinical features	LNM (+) group (n=43)	Non-LNM(-) group (n=254)	t/x^2^	P
Gender			0.026	0.872
male	25(58.14)	151(59.45)		
Female	18(41.86)	103(40.55)		
Age (years)	61.05±10.45	61.42±10.02	-0.225	0.822
Tumor site			2.357	0.308
upper	4(9.30)	13(5.12)		
central	19(44.19)	140(55.12)		
lower part	20(46.51)	101(39.76)		
Tumour size (mm)			9.192	0.002
≤20	24(55.81)	146(57.48)		
>20	29(67.44)	108(42.52)		
Gross typing			1.037	0.595
uplift	4(9.30)	38(14.96)		
flat	19(44.19)	110(43.31)		
Sag	20(46.51)	106(41.73)		
Infiltration depth			20.606	0.001
mucosal layer	22(51.16)	209(82.28)		
submucosa	21(48.84)	45(17.72)		
Vascular invasion			24.365	0.001
yes	22(51.16)	44(17.32)		
no	21(48.84)	210(82.68)		
Nerve invasion			16.908	0.001
yes	15(34.88)	28(11.02)		
no	28(65.12)	226(88.98)		
signet ring cell			0.459	0.498
Yes	4 9.30	33(12.99)		
no	39(90.70)	221(87.01)		
Differentiation type			5.315	0.021
differentiates	17(39.53)	157(61.81)		
undifferentiated	26(60.47)	97(38.19)		
Ulcer				
have	21(48.84)	101(39.76)		
none	22(51.16)	153(60.24)		
High NLR			0.213	0.644
yes	28(65.12)	156(61.42)		
no	15(34.88)	98(38.58)		
High PLR			0.473	0.492
yes	27(62.79)	173(68.11)		
no	16(37.21)	81(31.89)		
P53			7.045	0.008
feminine	6(13.95)	87(34.25)		
positivity	37(86.05	167(65.75)		
Ki67			0.069	0.792
feminine	4(9.30)	27(10.63)		
positivity	39(90.70)	227(89.37)		

### Multivariate analysis

In Table 1 above, parameters with P < 0.05 are taken as »independent variables«, and whether LNM (1=yes, 0= no) occurs in EGC patients after radical surgery is taken as »dependent variable« – specific values (Table 2). It was found by binary multivariate Logistic regression analysis, the relevant predictors of postoperative LNM in EGC patients were tumour size (OR: 2.582, 95% CI: 1.205–5.534), depth of invasion (OR: 2.953, 95% CI: 1.327–6.573), vascular invasion (OR: 2.724, 95% CI: 1.241–5.976), neuroaggression (OR: 2.681, 95% CI: 1.139–6.311), differentiation type (OR: 2.426, 95% CI: 1.140–5.119) and P53 (OR: 3.133, 95% CI: 1.183~8.301), P<0.05, as shown in Table 3.

**Table 2:** Variable assignment.

Independent variable	Variable description	Assignment
Tumor size	Categorical variables	≤20=0 >20
Infiltration depth	Categorical variables	Mucosal layer = 0, Submucosa = 1
Vascular invasion	Categorical variables	no -0, yes = 1
Nerve invasion	Categorical variables	no -0, yes = 1
Differentiation type	Categorical variables	differentiated=0, undifferentiated=1
P53	Categorical variables	feminine=0, positivity=1

**Table 3:** Multivariate Logistic regression analysis of LNM after EGC surgery.

Variable	B	SE	Wald	P	OR	95% CI
Tumor size	0.949	0.389	5.949	0.015	2.582	1.205~5.534
Infiltration depth	1.083	0.408	7.035	0.008	2.953	1.327~6.573
Vascular invasion	1.002	0.401	6.245	0.012	2.724	1.241~5.976
Nerve invasion	0.986	0.437	5.094	0.024	2.681	1.139~6.311
Differentiation type	0.882	0.383	5.298	0.021	2.426	1.140~5.119
P53	1.142	0.497	5.278	0.022	3.133	1.183~8.301

### Prediction effect

**Figure 3:**
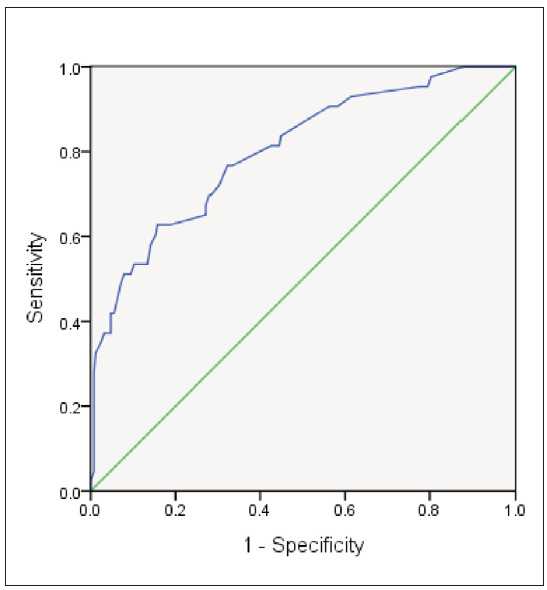
Comparison of LNM number between Non-LNM (-) group and LNM (+) group.

The Logistic regression prediction model for LNM after EGC radical surgery was established according to the relevant indexes in Table 3. The AUC of the ROC curve in the model was 0.801 (95% CI: 0.727–0.876), and the specificity and sensitivity were 67.70% and 76.70%, Figure 3.

### Follow-up analysis

After a 2-year follow-up, the OS rates were 88.37% at 1 year and 81.40% at 2 years in the LNM group, 99.61% at 1 year and 98.82% at 2 years in the non-LNM group. LNM group was significantly lower (all P<0.05); see Table 4.

**Table 4:** Comparison of survival between the two groups (%).

Group	1 year OS (%)	2 year OS (%)
LNM (+) group	38 88.37	35 81.40
non-LNM (-)	253 99.61	251 98.82
X^2^	23.448	31.302
P	0.001	0.001

## Discussion

The treatment and prognosis of early gastric cancer (EGC) have been the focus of recent research, with radical surgery remaining the primary treatment method. However, even when patients undergo radical surgery for gastric cancer, over 50% die due to tumour recurrence and metastasis [12]. Several studies [13] [14] have shown that the proportion of lymph node metastasis (LNM) in EGC patients is only 8.9% to 15.8%. This suggests that approximately 80% of patients who underwent endoscopic submucosal dissection (ESD) received unnecessarily extensive excision.

Gunji et al. [15] conducted a study on a cohort of patients following EGC surgery and found that the LNM rate was 10.2% during follow-up. The study also revealed a correlation between the number of LNMs and the patients’ postoperative prognosis, with a higher number of lymph nodes and more distant metastases being associated with poorer outcomes.

Therefore, an accurate preoperative evaluation of LNM is crucial for selecting appropriate and effective treatment options for EGC patients. In this study, 43 cases (14.48%) of 297 EGC patients developed LNM following radical surgery. The number of lymph nodes detected in the LNM group was higher, aligning closely with the findings of the study mentioned above.

The clinicopathological data of 297 EGC cases were collected in this study, and factors potentially influencing lymph node metastasis (LNM) were analysed individually. Binary multivariate logistic regression analysis revealed that the predictors significantly associated with LNM in EGC patients after radical surgery included invasion depth, tumour size, vascular invasion, differentiation type, and P53 expression (all P<0.05). These factors were further analysed as follows:

Tumour size is closely related to the ability of LNM [16]. As tumour volume increases, the likelihood of contact with the lymphatic system rises significantly, enhancing the probability of tumour infiltration into the submucosa and thereby promoting LNM [17]. According to the literature, LNM correlates with the depth of tumour invasion in EGC. When the lesion is confined to the mucosal layer, the risk of LNM is 3.0%–4.9%. However, when the submucosal layer is infiltrated, the risk increases to 23%–25% [18]. With continued infiltration into the interstitium and submucosa, tumour cells invade capillary lymphatic vessels and microvessels, significantly raising the LNM rate.

Sekiguchi et al. found that the LNM rate in cases with vascular invasion was 37.1%, markedly higher than the 5.7% observed in cases without vascular invasion [19]. Tumours accompanied by nerve invasion often lead to a poor prognosis, as nerve invasion is associated with severe back pain in advanced cases, reducing patients’ quality of life [20]. Reports also indicate that when EGC exhibits nerve invasion, and ulcers accompany the tumour surface, the LNM rate is higher. This may occur because ulcers compromise the submucosa, enabling the mucosal layer to connect more easily with the lymph node network in the submucosa, thereby promoting LNM [21].

However, some studies suggest nerve invasion is not associated with LNM [22]. This study observed no statistically significant relationship between ulceration and LNM, possibly due to the small sample size. Undifferentiated EGC is more likely to exhibit LNM than differentiated EGC [23]. Feng et al. [24] analysed 976 patients with EGC and reported an LNM incidence of 6.6% in differentiated cases, compared to 20.5% in undifferentiated cases. Similarly, Zhao et al. [25] identified mixed and undifferentiated types as major risk factors for LNM in EGC. Poorer differentiation is associated with increased tumour cell heterogeneity, a more invasive growth pattern, and a greater predisposition to LNM.

P53, a tumour suppressor gene, plays a crucial role in DNA-mediated apoptosis and cell cycle regulation. Studies indicate that mutations in the P53 gene contribute significantly to the progression of EGC to advanced gastric cancer and are closely associated with LNM [26]. Victorzon et al. [27] also found that high expression of the P53 protein is negatively correlated with the prognosis of gastric cancer patients. Numerous genetic, biochemical, physiological, and epigenetic findings are relevant to understanding and managing gastric cancer [28] [29] [30] [31] [32] [33] [34] [35] [36] [37] [38] [39] [40] [41] [42] [43] [44] [45].

In the Logistic regression prediction model of LNM after EGC was established in this study, the ROC curve AUC was 0.801, the specificity was 67.70%, and the sensitivity was 76.70%. It can be seen that the prediction effect of LNM occurrence based on clinically relevant detection indexes and pathological P53 indexes of patients after EGC radical surgery is better. In this paper, patients who had undergone radical resection of EGC were followed up for 2 years. The results showed that patients with LNM had lower overall survival rates at 1 and 2 years than the non-LNM. It can be seen that improving the prediction accuracy of LNM in patients with EGC has great clinical significance in improving their survival time. Considering the small sample size of this study, further data collection is needed to confirm whether the degree of differentiation guides individual treatment decisions for patients with EGC.

## Conclusion

This study was conducted to address the critical need for effective preoperative prediction of lymph node metastasis in early gastric cancer, as lymph node metastasis significantly impacts prognosis and survival. By analysing serum markers (NLR, PLR) and pathological factors (P53, Ki67, tumour size, invasion depth, vascular invasion, and nerve invasion), we aimed to identify reliable predictors of LNM. Our findings demonstrate that these factors, particularly P53 expression and tumour characteristics, have significant predictive value, as evidenced by the logistic regression model (AUC: 0.801). This model provides a practical tool to aid clinicians in tailoring treatment strategies and improving patient outcomes. Addressing the study’s objective, we show that combining molecular and pathological markers offers a robust method for risk stratification, enabling early intervention. Further multicenter studies are encouraged to validate these findings and enhance their clinical applicability.

## Dodatak

### Acknowledgements

We sincerely thank the Sichuan Integrative Medicine Hospital and the Oncology Center, School of Medicine, UESTC, for providing the resources and data necessary for this study. We also thank our colleagues from the Department of Medical Oncology and other collaborators who contributed to this research.

### Author contributions

Xin Long: Conceptualization, Data curation, and Statistical analysis.

Yingxi Shi: Methodology and Writing – original draft.

Feifei Li: Data curation and Literature review.

Zhaojun Wang: Validation and Supervision.

Ying Wang: Writing – review and editing, Supervision, and Project administration.

All authors have read and approved the final manuscript.

### Ethical approval

The Institutional Review Board of Sichuan Integrative Medicine Hospital approved this study. All procedures were conducted following the ethical standards of the Helsinki Declaration. Written informed consent was obtained from all participants before data collection.

### Data availability statement

The datasets used and analysed during the current study are available from the corresponding author upon reasonable request.

### Conflict of interest statement

All the authors declare that they have no conflict of interest in this work.
